# X-Ray Shielding Polymer Based on Sequential Polycondensation of BiPh_3_ and Carboxylic Acids and Radical Polymerization

**DOI:** 10.3390/polym17020134

**Published:** 2025-01-08

**Authors:** Bungo Ochiai, Ryo Kamiya, Yoshimasa Matsumura, Hiroyasu Tanaka, Hideki Ueda, Kazuyoshi Uera, Kikuo Furukawa, Yoshio Nishimura

**Affiliations:** 1 Graduate School of Science and Engineering, Yamagata University, Yonezawa 990-8510, Japan; 2 Department of Applied Chemistry, Faculty of Engineering, Osaka Institute of Technology, Osaka 535-8585, Japan; yoshimasa.matsumura@oit.ac.jp; 3 Mitsubishi Gas Chemical Company, Inc., Tokyo 100-8324, Japan

**Keywords:** bismuth, organic–inorganic hybrid, optical material, X-ray shielding, refractive index

## Abstract

Transparent X-ray shielding polymer films were developed by bulk photo copolymerization of in situ prepared bismuth carboxylate prepolymers with polymerizable exomethylene moieties and *N*,*N*-dimethylacrylamide (DMAA). The bismuth-containing prepolymers were prepared via the polycondensation of BiPh_3_, 2-octenylsuccinic acid (OSA), and itaconic acid (IA) bearing an exomethylene group for polymerization. OSA was a chain extender by intermolecular condensation and a stopper by intramolecular cyclization to inhibit cross-linkage. The resulting photocured films exhibit high visible-light transparency and high *n*_D_, reaching 1.57. The X-ray shielding ability increased with the bismuth content and reached an aluminum equivalent of 0.80.

## 1. Introduction

Radiation plays essential roles in various fields, such as diagnosis, instrumental analyses, and power generation, while adequate shielding is mandatory due to its effects on the human body and the environment. Gamma rays and X-rays are highly penetrating forms of radiation, and their handling with visibility requires the use of transparent protective materials to safeguard the eyes, which are susceptible to radiation exposure. Typical radiation shielding goggles are made of inorganic glasses or organic resins containing lead oxide [1,2,3,4]. Resin-based materials are used for comfortable protective eyewear due to their superior processability and light weight, although they have inferior shielding properties. However, lead is toxic and has adverse effects on the human body and the environment [5]. Consequently, regulations have been tightened, and the use of other metals as lead substitutes is mandatory [6,7,8,9,10,11,12,13,14,15]. Barium [7], copper [3], and titanium [8,9,10], for example, are safer alternatives but exhibit inferior radiation shielding properties. Cadmium [11] and antimony [12] have superior radiation shielding properties but are not viable solutions due to their toxicity. Tungsten [13,14,15] has low toxicity and good radiation shielding properties but is costly. Therefore, the development of materials with radiation shielding and safety is essential to protect workers and the environment relating to radiation industries.

Bismuth is a promising alternative due to its status as the element with the highest atomic weight among those utilized in daily life, which provides the high radiation-shielding characteristic of heavy metals. An additional benefit of bismuth is its low toxicity. Many bismuth compounds have low toxicity; for instance, the L_D50_ of BiCl_3_ for mice is greater than that of NaCl [16]. Therefore, safe X-ray shielding materials have been developed using bismuth oxide nanoparticles or alternative bismuth compounds as fillers [17,18,19,20]. For example, Yu et al. reported a composite material containing bismuth titanate nanoparticles in an epoxy resin matrix [19]. The resulting material exhibited a significant enhancement in X-ray shielding properties of epoxy resins, which was further augmented with an increase in the weight ratio of bismuth oxide nanoparticles. Although this material is suitable for X-ray protective clothing, it is not appropriate for protective eyewear due to the lack of transparency.

For the sake of transparency, it is necessary to compound bismuth at the molecular level with organic materials such as polymers. Arylbismuthine and bismuth carboxylate structures are typical frameworks for stable complexation [21,22,23,24,25,26,27,28]. Covalent bismuth-containing polymers with aryl—bismuth bonds are synthesized by the polymerization of styrylbismuthines [21,22,23,24]. The radiation-shielding abilities of these polymers are superior to those of bismuth carboxylate polymers. However, the synthetic procedures are more laborious than those for bismuth carboxylates, and the yellowish color deteriorates the visibility. Synthesizing bismuth carboxylate polymers is a more straightforward method [25,26,27,28]. The copolymerization of bismuth methacrylate oxide clusters with methyl methacrylate (MMA) in DMF, with the subsequent removal of DMF, yields a semi-transparent grayish X-ray shielding polymer with a bismuth content of 22.5 wt% [25]. The X-ray shielding properties of the obtained polymers increased with the bismuth content and reached up to the radiopacity equivalent to that of a 2.3 mm thickness of aluminum. However, the thickness of the pellets measured was not disclosed. Our group has previously reported the preparation of bismuth-containing polymer films with high transparency by the bulk copolymerization of bismuth methacrylate (BiMA), which was produced by the nucleophilic substitution of BiPh_3_ and methacrylic acid, and *N*,*N*-dimethylacrylamide (DMAA) [27]. However, due to the low solubility of BiMA, the maximum bismuth content in the films was limited to 18 wt%, and the X-ray shielding property at the Al equivalent was 0.60. The replacement of the polymerizable carboxylate moieties with those of the acrylic acid dimer resulted in the increase in the bismuth content of the resulting polymer (poly(BiCEA-*co*-2CEA)) to 30.7 wt% due to its high solubility [28]. As a result, the X-ray shielding property at the Al equivalent was enhanced up to 1.31 despite the yellowed color.

As a strategy to overcome the low bismuth content due to low solubility, which is a challenge for bismuth carboxylate polymers, we propose the copolymerization of a vinyl monomer and polymerizable bismuth polymers prepared by the in situ polycondensation using dicarboxylic acids for radical polymerization and chain extension. This research revealed the synthetic method for curing organic–bismuth hybrid films with high transparency and refractive indices, as well as X-ray shielding abilities comparable to those of light metals, which are high as colorless transparent polymers.

## 2. Materials and Methods

### 2.1. Materials

BiPh_3_ was prepared as reported [29]. 2-Octenylsuccinic acid (OSA) was prepared via the hydrolysis of 2-octenylsuccinic acid as reported (Figures S1 and S2) [30]. Itaconic acid (IA), 2-octenylsuccinic acid, and suberic acid were purchased from Tokyo Chemical Industry (Tokyo, Japan). Methanol, hexane, dehydrated tetrahydrofuran (THF), and dehydrated methyl ethyl ketone (MEK) were purchased from Kanto Chemical (Tokyo, Japan). DMAA was purchased from Wako Pure Chemical (Tokyo, Japan). 2,4,6-Trimethylbenzoyl-diphenyl-phosphineoxide (Darocur TPO) was purchased from Ciba Specialty Chemicals (Basel, Switzerland).

### 2.2. Measurements

^1^H NMR spectra were measured on a JEOL (Tokyo, Japan) ECX-400 spectrometer using CDCl_3_ or *d*_6_-dimethylsulfoxide (DMSO) as the solvent (23–25 °C, relaxation delay = 5 s, 8 scans). The residual solvent signals of CHCl_3_ or *d*_5_-DMSO were used as the internal reference. Fourier transform infrared (FTIR) spectra were measured on a JASCO (Tokyo, Japan) FT/IR-460Plus spectrometer equipped with a JASCO ATR PRO400-S attenuated total reflectance accessory and a ZnSe prism. Dynamic light scattering (DLS) measurements were carried out using a Malvern (Worcestershire, UK) Zetasizer nano-ZS instrument equipped with a 4 mW He-Ne laser (633 nm) and 12 mm square glass cuvettes at 25 °C. Thermogravimetric analysis (TGA) was conducted using a Seiko Instruments (Chiba, Japan) EXSTAR 6000 TG/DTA 6200 instrument under a nitrogen atmosphere at a heating rate of 10 °C/min. Differential scanning calorimetry (DSC) measurements were performed on a Seiko Instruments DSC-220 instrument under a nitrogen atmosphere (10 °C/min, N_2_, with a second heating scan after heating to 100 °C and cooling to −80 °C at 10 °C/min). UV-vis absorption and transmittance spectra were recorded on a Shimadzu (Kyoto, Japan) UV-2600 spectrometer equipped with a Shimadzu ISR-2600 integral sphere to minimize the effects of diffusion and scattering. Scanning electron microscope (SEM) measurements were conducted on a JEOL (Tokyo, Japan) JSM-6510A microscope at an accelerating voltage of 10 kV. Energy dispersive X-ray (EDX) analysis was performed with a system consisting of a JEOL JSM6510A scanning electron microscope equipped with a JEOL JED2300 EDX spectrometer operated at an acceleration voltage of 15 kV. Refractive indices (*n*_D_s) were measured with an Atago (Tokyo, Japan) DR-A1 digital Abbe refractometer. X-ray transmittance ratios of Cu *K*α (8.0 keV, acceleration voltage = 40 kV, current = 30 mA, irradiation time = 10 min) were evaluated using a Rigaku (Tokyo, Japan) Nanoviewer equipped with a Dectris (Baden, Switzerland) PILATUS-100k X-ray detector. The X-ray transmittance was measured from the ratios of total counts of direct beam and that attenuated by samples.

### 2.3. Polycondensation of BiPh_3_ and OSA (Typical Procedure)

BiPh_3_ (220 mg, 0.500 mmol) and OSA (114 mg, 0.500 mmol) were added in a two-necked test tube and dissolved in dehydrated MEK (1.0 mL). The mixture was refluxed for 24 h while stirring. Then, the resulting solution was poured into a 50 mL amount of hexane to precipitate the resulting product. The white solid was separated by centrifugation and dried under reduced pressure (202 mg, 79.3%). ^1^H-NMR (400 MHz, CDCl_3_, *δ* in ppm): 0.76–0.99 (br, 3H, –CH_2_C*H*_3_), 1.12–1.53 (br, 6H, –CH_2_C*H*_2_C*H*_2_C*H*_2_CH_3_), 1.66–3.94 (br, 7H, =CHC*H*_2_CH_2_–, >CHC*H*_2_CH=, –OCOC*H*_2_C*H*<), 5.01–5.79 (br, 2H, –CH_2_C*H*=C*H*CH_2_–).

### 2.4. Polycondensation of BiPh_3_, OSA, and IA (Typical Procedure)

BiPh_3_ (881 mg, 2.00 mmol), OSA (411 mg, 1.80 mmol), and IA (26.0 mg, 0.200 mmol) were added in a two-necked test tube and dissolved in dehydrated THF (1.0 mL). The mixture was refluxed for 24 h while stirring. Then, the resulting solution was poured into a 50 mL amount of hexane to precipitate the resulting product. White solid was separated by centrifugation and drying under reduced pressure (824 mg, 90.1%). ^1^H-NMR (400 MHz, CDCl_3_, *δ* in ppm): 0.74–0.98 (br, 3 × 0.924H, –CH_2_C*H*_3_), 1.00–1.53 (br, 6 × 0.924H, –CH_2_C*H*_2_C*H*_2_C*H*_2_CH_3_), 1.60–3.92 (br, 7 × 0.924 and 2 × 0.076H, =CHC*H*_2_CH_2_–, >CHC*H*_2_CH=, –OCOC*H*_2_C*H*<, –OC(=O)C*H*_2_C(=CH_2_)COO–), 4.91–5.94 (br, 2 × 0.924 and 1 × 0.076H, –CH_2_C*H*=C*H*CH_2_– and *Z*-*H*_2_C=C<), 6.04–6.69 (br, 1 × 0.076H, *E*-*H*_2_C=C<).

### 2.5. Polycondensation of BiPh_3_, OSA, and SA (Typical Procedure)

BiPh_3_ (220 mg, 0.500 mmol), OSA (79.9 mg, 0.350 mmol), and SA (26.1 mg, 0.150 mmol) were added in a two-necked test tube and dissolved in dehydrated THF (1.0 mL). The mixture was refluxed for 24 h while stirring. Then, the resulting solution was poured into a 50 mL amount of hexane to precipitate the resulting product. White solids with poor solubility were separated by centrifugation and drying under reduced pressure (144 mg, 72.4%).

### 2.6. Photocuring of Bismuth Carboxylate Polymer In Situ Prepared by the Polycondensation of BiPh_3_, OSA, and IA in DMAA

BiPh_3_ (220 mg, 0.500 mmol), OSA (79.9 mg, 0.350 mmol), and IA (19.5 mg, 0.150 mmol) were added in a two-necked test tube and dissolved in DMAA (1.0 mL). The mixture was heated at 70 °C for 24 h while stirring. Then, DMAA was partially evaporated under reduced pressure. The composition of DMAA and the other components was determined by ^1^H NMR spectroscopic analysis, in which the integral ratios of the vinyl protons of DMAA and methyl protons of the alkyl chain of the OSA unit were compared. TPO (1.0 mol% to total molar amounts of DMAA and IA) was added to the mixture, and the resulting liquid was cured in a mold consisting of two glass plates with fluorine coating using a Fine Chemical Japan (Tokyo, Japan) FC-250 mold release agent sandwiching a Teflon spacer by irradiation of 365 nm UV light using a CCS (Kyoto, Japan) HLDL-100U6-4UPSC LED light for 10 min. IR (Bi12(OSA-IA:7-3)-*co*-DMAA, cm^−1^): 621, 694, 715, 728, 757, 792, 874, 971, 1056, 1092, 1138, 1210, 1257, 1351, 1397, 1417, 1451, 1496, 1630, 1724, 2855, 2921, 3470.

## 3. Results

### 3.1. Optimization of Synthesis of Bismuth Carboxylate Prepolymers

**Scheme 1:**
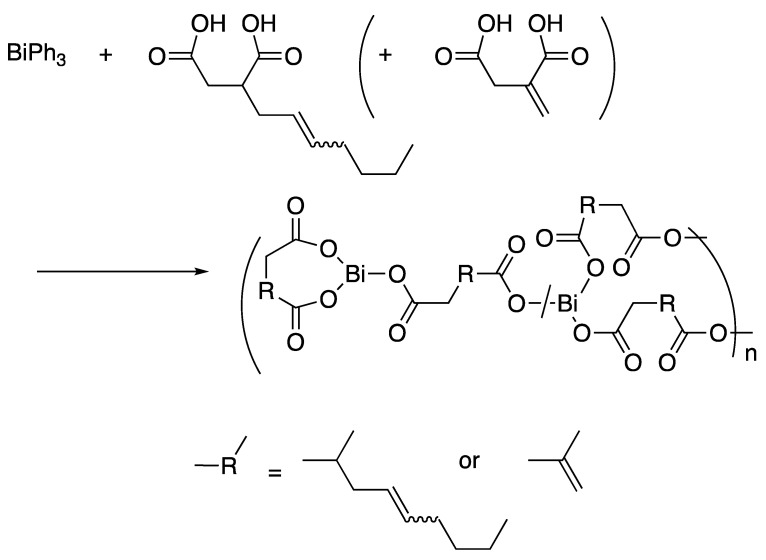
Polycondensation of BiPh_3_ and dicarboxylic acids.

OSA, which is a readily available succinic acid derivative from a commercial acid anhydride [30], was used as the dicarboxylic acid. The octenyl chain confers solubility to the polymer. The succinic acid moieties were selected to balance the composition of branch and terminating units since condensation of dicarboxylic acids and trivalent bismuth leads to cross-linked polymers without appropriate termination. The terminating unit was produced by intramolecular cyclization as reported for reactions of certain succinic acid derivatives with BiPh_3_ that yield cyclic bismuth carboxylates [31]. Polycondensation of BiPh_3_ with different molar ratios of dicarboxylic acids was carried out in 1 M solution for 24 h (Scheme 1, Table 1). White solids were obtained in good yields under all conditions. The structural analysis of the products obtained is described in the following section. Initially, the reaction conditions were optimized for polymerization with OSA. Polymerization in THF gave a homogeneous solution after the reaction (Run 1), whereas polymerization in MEK yielded a heterogeneous dispersion (Run 2). Accordingly, subsequent polymerizations were conducted in THF. The precipitation and purification with hexane as a poor solvent gave soluble products, whereas precipitation and purification with methanol gave insoluble products, which is likely due to hydrolysis. The effect of the feed ratios of OSA to BiPh_3_ was examined (Run 2–4). THF-soluble products were obtained using 1.0 and 1.2 equivalents of OSA. In contrast, polymerization with 1.5 equivalents of OSA gave a product insoluble in common organic solvents. This difference may be attributed to the predominance of intermolecular cross-linking over intramolecular cyclization as the ratio of OSA increases, which results in insolubility. These findings illustrate that controlling the ratio of dicarboxylic acids in the appropriate reaction solvent is crucial for obtaining soluble products.

**Figure 1:**
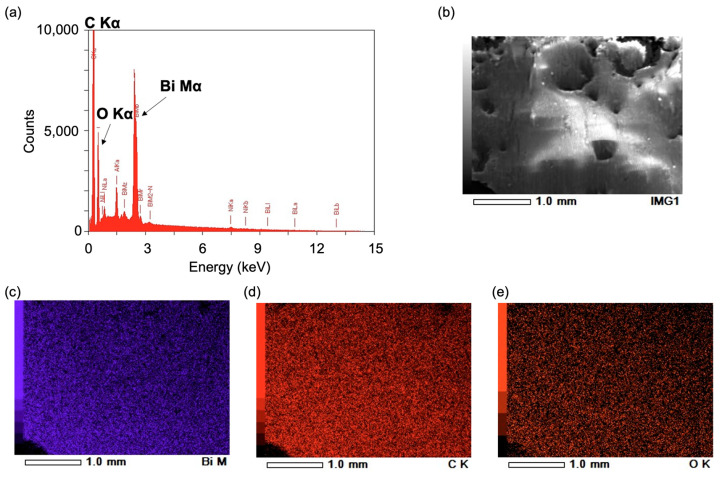
(**a**) EDX spectrum, (**b**) SEM image, and elemental maps [(**c**) Bi, (**d**) C, and (**e**) O] of bismuth carboxylate polymer obtained in Run 2 in Table 1.

**Figure 2:**
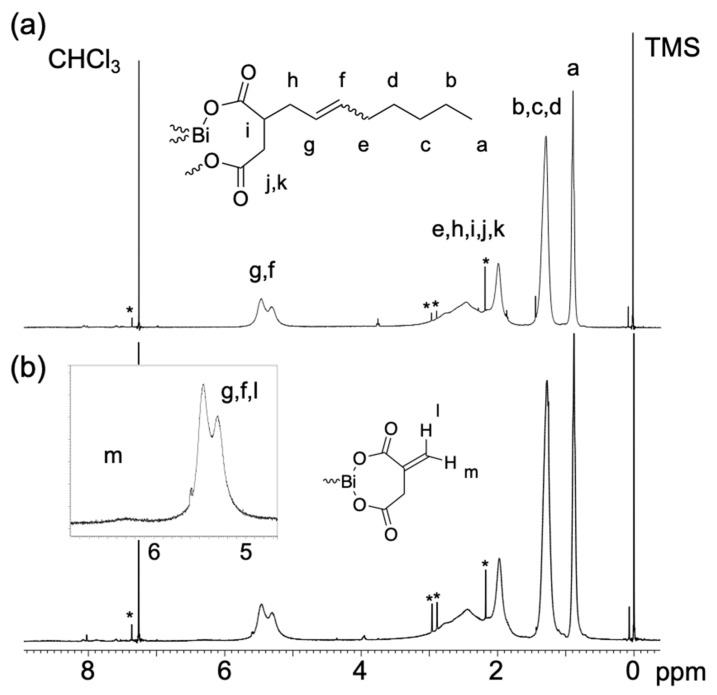
^1^H-NMR spectra of bismuth carboxylate polymers obtained in Run (**a**) 2 and (**b**) 7 in Table 1 (CDCl_3_, 400 MHz). Asterisks indicate the signals of residual acetone and *N*,*N*-dimethylformamide used to wash NMR tubes.

The structural analysis of the product obtained in Run 2 is shown as an example. The EDX spectrum and elemental maps demonstrated the presence and homogeneous distribution of Bi, C, and O (Figure 1). The ^1^H-NMR spectrum (Figure 2a) showed broadened peaks at approximately the same positions as observed in the spectrum of OSA (Figure S1). This broadening was more pronounced in the peaks i′, j′, and k′ at 1.8–3.5 ppm, which are assigned to the protons in proximity to the carboxylic acid structure. By contrast, the peaks originating from the terminal alkyl group in the octenyl chain (0.8–0.9 ppm for C*H*_3_–, 1.2–1.4 ppm for CH_3_C*H*_2_C*H*_2_C*H*_2_–, and 1.9–2.0 ppm for BuC*H*_2_–) and alkenyl groups (5.1–5.8 ppm) were sharper. No peaks derived from the carboxylic acid protons were observed. This observation supports the formation of the bismuth carboxylate structure, while the broad and overlapped peaks did not allow the calculation of ratios of the branch and terminating units [27,28,31]. On the other hand, the intensity of the Bi–Ph group peak at 7.0–8.0 ppm is considerably lower than that estimated from the feed ratio. This discrepancy suggests the hydrolysis of Bi–Ph bonds during the purification process or the removal of the bismuth complexes containing a greater number of terminal Ph groups, which are anticipated to have lower molecular weight.

**Figure 3:**
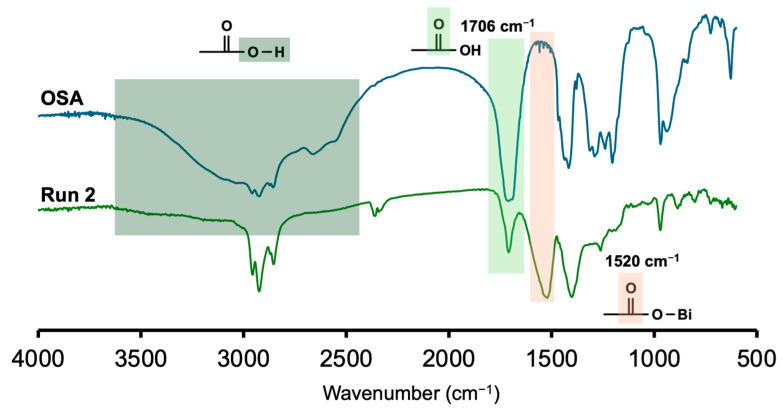
FT-IR spectra of bismuth carboxylate polymer obtained in Run 2 in Table 1 and OSA (ATR).

The FT-IR spectrum (Figure 3) displays a characteristic peak attributed to the carbonyl group in bismuth carboxylate at 1520 cm^−1^ [27,28], which is absent in the spectrum of crude OSA. Similar low-wavenumber shifts occur for various metal succinate derivatives [32,33,34,35]. Additionally, a weak peak, which was attributed to the carbonyl group of the carboxylic acid, was observed at 1706 cm^−1^. On the other hand, a peak at 2500–3000 cm^−1^ derived from the O–H of the carboxylic acid is not observed. This carboxylic acid may be present only in a small amount at the end or may have been formed by hydrolysis during the measurement.

The molecular size was estimated by DLS measurements in THF by virtue of the solubility in THF. This approach was employed because the hydrolytic instability inhibited the molecular weight analysis by size exclusion chromatography. The hydrodynamic diameter (*D*_h_) was 27 nm, and the polydispersity index (PDI) was slightly broader, 0.088. These values suggest the presence of soluble branched polymers and partially cross-linked nanogels. These spectroscopic and DLS measurement results demonstrate the formation of bismuth-containing branched polymers, as illustrated in Scheme 1.

In view of these results, copolymers bearing polymerizable groups were synthesized (Runs 5, 6, and 7 in Table 1). IA was selected as the dicarboxylic acid for complexation and polymerization [34,35]. Copolymerization was carried out with different ratios of BiPh_3_, OSA, and IA. The polymerization at a feed molar ratio of OSA:IA = 7:3 (Run 5) yielded soluble and insoluble products. The soluble product was so unstable that it became insoluble when THF was removed, and thus, its yield could not be accurately determined. The polymerization (Run 6) with an OSA:IA ratio of 8:2 yielded a homogeneous solution. However, the product became insoluble upon purification by precipitation in hexane. In contrast, the polymerization with a feed ratio of OSA:IA = 9:1 (Run 7) yielded a product that retained the solubility after the purification by precipitation. The structure of this product was evaluated by ^1^H-NMR spectroscopy (Figure 2b). A broad peak was observed at 6.0–6.7 ppm, which corresponds to the region of the exomethylene proton of IA far from the bismuth carboxylate bond [32]. This peak was not observed in the spectrum of the product obtained only with OSA. This peak indicates that the structure derived from IA was introduced into the product. The signal of the other proton overlaps with that of the alkenyl group in the octenyl chain [32]. The copolymer composition was estimated to be OSA:IA = 92:8 based on the integral ratio of the peaks derived from the –CH_2_CH=CHCH_2_– group (g and f) in the OSA unit overlapping with one exomethylene proton in the IA unit (l) and the peak derived from the exomethylene proton (m) in the IA unit. However, the low intensity of this IA-derived peak is insufficient for accurate estimation.

To confirm the effectiveness of intramolecular cyclization, polymerization was performed with SA, where a long spacer between the two carboxylic acid moieties strongly favors intermolecular condensation over intramolecular cyclization (Runs 8 and 9 in Table 1). This insolubility is due to the presence of SA. Polymerization with a higher OSA ratio of OSA:SA = 7:3 (Run 9) yielded a homogeneous solution. However, following precipitation purification, the product became insoluble, probably due to the low hydrolytic resistance of the linear and cross-linked bismuth carboxylate structures that were inherently formed. These results indicate that the intramolecular cyclization with OSA effectively suppressed cross-linking and rendered the products sufficiently stable enough for this polycondensation system. However, none of the polymers obtained could be processed into transparent films, and an alternative approach was therefore pursued, capitalizing on the advantage of the polymerizability of the IA units.

### 3.2. Synthesis and Properties of Bix(OSA-IA:y-z)-co-DMAA

**Scheme 2:**
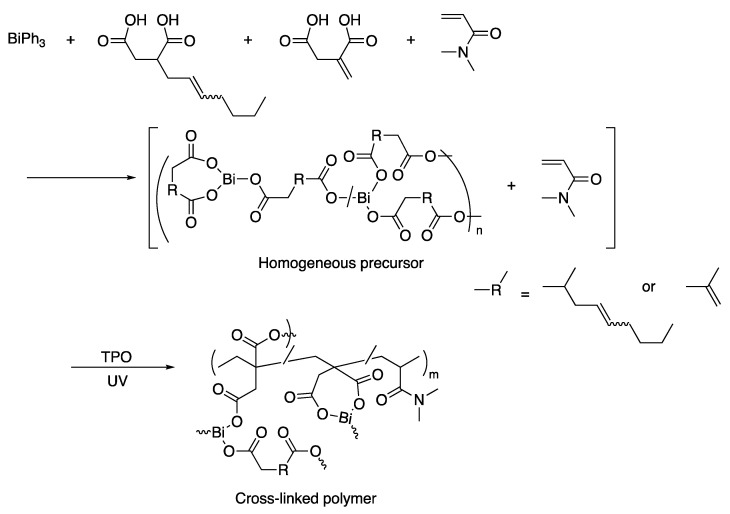
Photo curing of DMAA and bismuth carboxylate polymer in situ prepared by polycondensation of BiPh_3_ and dicarboxylic acids.

Polymerizable bismuth carboxylate polymers were prepared by polycondensation in DMAA and subsequently cured to produce bismuth-containing cross-linked polymer films (Scheme 2). The first process was polycondensation of BiPh_3_ with a 0.7 equivalent of OSA and a 0.3 equivalent of IA in DMAA (2 M) at 70 °C for 24 h. The second process was photocuring after concentration by evaporation of DMAA under reduced pressure. The composition of each component in the concentrated, viscous product was determined by ^1^H-NMR spectroscopy. Subsequently, TPO (1 mol% of the total amount of DMAA and IA) was added as an initiator. The photocuring was conducted by irradiating the homogeneous precursor with 365 nm UV light for 10 min, resulting in the formation of transparent films. The resulting bismuth-containing film was designated as (Bix(OSA-IA:y-z)-*co*-DMAA), where x represents the molar fraction of bismuth to DMAA and y:z denotes the OSA:IA ratio.

**Figure 4:**
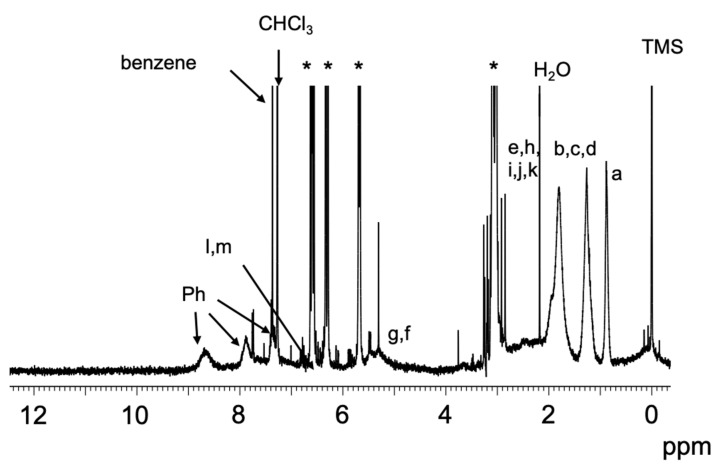
^1^H-NMR spectrum of precursor consisting of in situ-prepared bismuth carboxylate polymer and DMAA (CDCl_3_, 400 MHz). Asterisks indicate the signals of DMAA. The alphabetical assignments are the same as those in Figure 2.

As an example, the ^1^H NMR spectrum of a precursor comprising an in situ-prepared bismuth carboxylate polymer and DMAA with a Bi:DMAA molar ratio of 7:93 is presented in Figure 4. This ratio was determined from the integral ratio of the peaks of the vinyl protons of DMAA (B) and the peak of the methyl protons of the OSA backbone (a). The ^1^H-NMR spectrum of the concentrated mixture exhibited a broad peak derived from the OSA skeleton, as those observed in the spectra of mixtures resulting from polycondensation in THF. However, the alkenyl peak of the IA unit at 6.0–6.9 ppm was not clearly detected due to its overlap with the larger sharp peaks of the vinyl protons in DMAA. The molar compositions of eliminated benzene, unreacted BiPh_3_, and phenyl groups attached to bismuth carboxylate structures, which remained after vacuum concentration, were calculated to be 0.75, 0.18, and 2.9 mol%, respectively, relative to DMAA. However, the composition of the phenyl groups is relatively low in comparison to the feed ratio. A potential explanation for this low phenyl content is the hydrolysis of structures containing Bi-Ph groups by residual moisture in DMAA.

**Figure 5:**
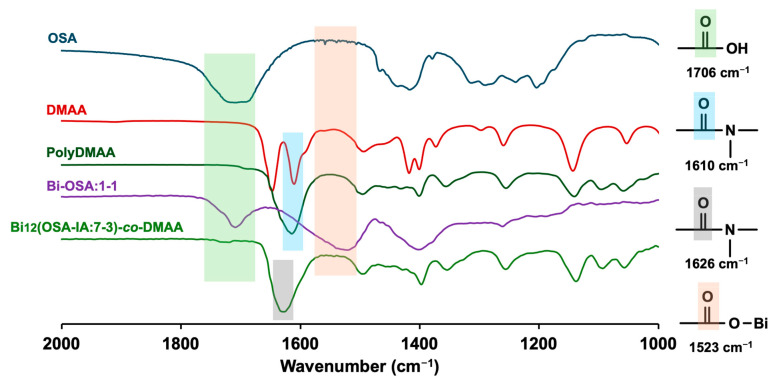
FT-IR spectra (1000–2000 cm^−1^) of Bi12(OSA-IA:7-3)-*co*-DMAA and precursors (ATR).

The FT-IR spectrum of Bi12(OSA-IA:7-3)-*co*-DMAA obtained by photocuring (Figure 5) exhibited the absence of absorption of the C—H stretching vibration of the vinyl group of DMAA at 3096 cm^−1^, which corroborates the progress of polymerization. The absorption of the carbonyl stretching vibration was observed as a broad peak consisting of peaks of bismuth carboxylate around 1523 cm^−1^ [27,28] and polyDMAA around 1613 cm^−1^. Additionally, the coordination of the amide carbonyl to the Lewis acidic bismuth carboxylate structure may also result in a low wavenumber shift, thereby broadening the peak. The presence of the bismuth carboxylate structure was also supported by the negligible peaks of the carbonyl (1706 cm^−1^) and O–H (2500–3300 cm^−1^) groups in the carboxylic acid group formed by hydrolysis.

The effect of varying the OSA:IA ratios was then investigated for a polymer with a Bi weight fraction of approximately 20% (Table 2). Bismuth carboxylate prepolymers with the IA units soluble in DMAA were obtained irrespective of the ratio of IA. Films could be prepared by subsequent concentration and photocuring. However, at an OSA:IA ratio of 3:7, the viscous mixture after concentration was insoluble in CDCl_3_, and the composition was determined from the ^1^H-NMR spectrum measured using *d*_6_-DMSO as the solvent. This different solubility is attributable to the IA units lacking flexible chains, which reduces solubility. All the photocured films were transparent, and their FT-IR spectra were similar to those described above (Figure S3).

All Bix(OSA-IA:y-z)-*co*-DMAA) were partially insoluble in THF, which is a good solvent for polyDMAA, and their gel fractions were determined by Soxhlet extraction with THF. The ^1^H-NMR spectra of the soluble fractions exhibited broad peaks assigned to Bi-OSA and polyDMAA structures. In contrast, no peaks originating from the exomethylene groups of IA were observed at 5.7–6.9 ppm (Figure S4). This result also supports the formation of a cross-linked structure by copolymerization of polymers bearing a bismuth carboxylate structure and DMAA through the IA unit. The increase in the IA composition resulted in the higher *T*_g_ and gel fraction due to the increased cross-linking efficiency (Figure S6). Thermal stability was evaluated from the 5%-weight-loss temperatures (*T*_d5_s). The *T*_d5_s were approximately 190 °C and increased with the IA content, which aligns with the increase in the cross-linking density. The thermal stability is inferior to that of polyDMAA but sufficient for typical applications.

Bix(OSA-IA:7-3)-*co*-DMAA films with different bismuth contents were prepared by varying the DMAA composition. The FT-IR spectra of the resulting films exhibited peaks at identical wavenumbers, while the spectra of the polymers with higher bismuth content demonstrated stronger absorption peaks of the carbonyl group of bismuth carboxylate around 1515–1595 cm^−1^ (Figure S5).

The bismuth contents mentioned above were determined by NMR spectroscopy and compared with those calculated from TGA. The plausible residue at 500 °C is Bi_2_O_3_, and the bismuth contents were calculated based on this presumption (Table 3). The bismuth contents calculated by NMR spectroscopy and TGA agreed well, indicating the validity of the calculation. The bismuth content had a negligible effect on *T*_g,_ probably due to the balance of the cross-linkage of the IA unit, which restricts the motion of polymer chains, and the flexible OSA side chains, which enhance mobility (Figure S7).

**Figure 6:**
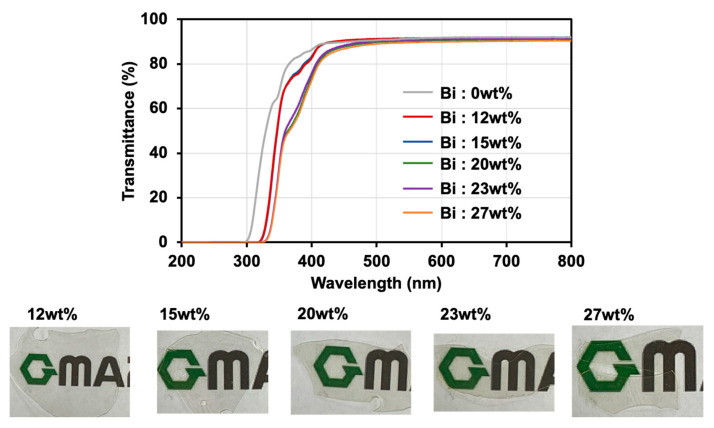
Transmittance spectra and photo images of Bix(OSA-IA:7-3)-*co*-DMAA films with different Bi contents.

**Figure 7:**
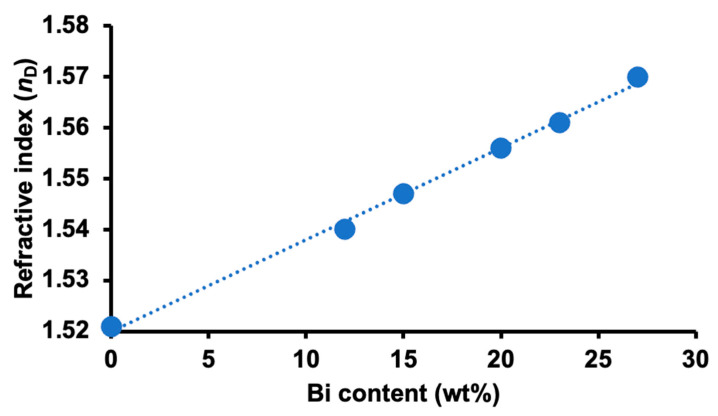
Relationship between Bi content (x) and *n*_D_ of Bix(OSA-IA:7-3)-*co*-DMAA.

Each film obtained was yellow in color immediately after photocuring. Conversely, the films became almost colorless and transparent, with a D line (589 nm) transmittance of approximately 90% after approximately one week of storage in the dark (Figure 6). The transparency of these polymer films is greater than that of previously reported bismuth polymers [21,22,23,24,25,26,27,28]. This coloration and subsequent decoloration may be attributed to either the reoxidation of colored Bi(0) species, which is produced by the photoreduction reported in the polymerization of bismuth methacrylate [27], to colorless Bi (III) species, or to the decomposition of initiator-derived colored substances. Similarly, reversible color changes due to redox of Bi species were also reported [36,37]. The refractive index (*n*_D_) of the film increased with an increase in bismuth content, reaching a maximum value of 1.57 at the highest bismuth content of 27 wt%. This refractive index is higher than that of poly(BiMA-*co*-DMAA), with the highest Bi content of 18.0 wt% (*n*_D_ = 1.55) in our previous report [27], due to the higher bismuth content (Figure 7). The *n*_D_s of the different Bix(OSA-IA:x-y)-*co*-DMAA films with Bi content of approximately 20 wt% were 1.56, irrespective of the organic components. This suggests that the bismuth content exerts a dominant influence on the refractive index rather than differences in the organic structure.

### 3.3. X-Ray Shielding Properties of Bix(OSA-IA:7-3)-co-DMAA

**Figure 8:**
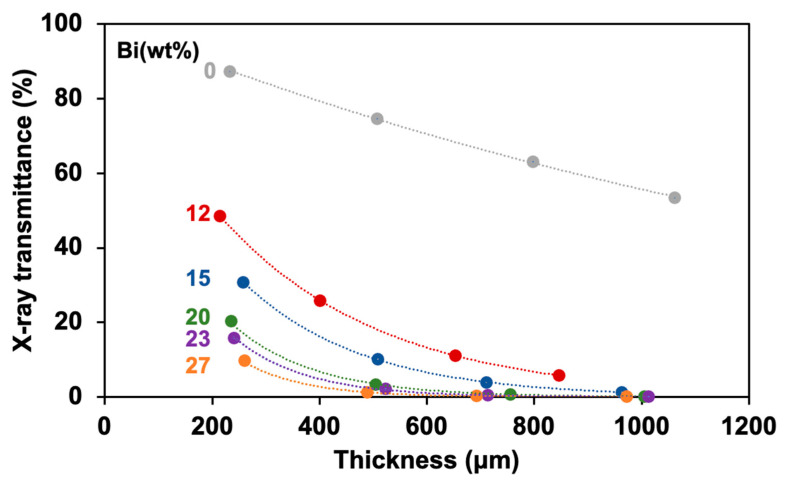
X-ray shielding properties of Bix(OSA-IA:7-3)-*co*-DMAA (Cu Kα (8.0 keV), acceleration voltage = 40 kV, irradiation time = 10 min).

The X-ray shielding properties of Bix(OSA-IA:7-3)-*co*-DMAA were evaluated for films of varying thicknesses (Figure 8, Table S1). It should be noted that this method underestimates the X-ray shielding properties of copper and aluminum at 3% and 19%, respectively. Accordingly, the data presented here may also be underestimated [27,28]. As the thickness of the films and the compositions of bismuth increase, the X-ray transmittance decreases, indicating an improvement in X-ray shielding properties. The film with the highest bismuth content of 27 wt%, Bi27(OSA-IA:7-3)-*co*-DMAA with a thickness of 723 μm, demonstrated an X-ray transmittance of 0.2%, effectively shielding X-rays. It should be noted that the thicknesses of lead and aluminum that give 0.2% X-ray transmittance at the same energy are 24 μm and 566 μm, respectively.

The X-ray shielding properties of the resulting bismuth-containing polymer and other materials were compared in terms of aluminum equivalent (Table 4). The aluminum equivalent of the bismuth-containing film was markedly higher than that of polyDMAA without containing bismuth. The Bi27(OSA-IA:7-3)-*co*-DMAA film with the highest bismuth composition exhibited an aluminum equivalent of 0.80 mm-Al/mm-polymer. This radiopacity is inferior to that of styrylbismuthine polymers [22,24], poly(BiCEA-*co*-2CEA) [28], and composites of inorganic nanoparticles of bismuth compounds and polymers [17,18]. The ionic linkages of bismuth carboxylate bonds, which reduce density, are responsible for the weaker attenuation abilities. However, the transparency of Bix(OSA-IA:7-3)-*co*-DMAA is significantly superior to that of these polymers. The materials without evaluation of transmittance [17,22] were not designed for transparent materials. For transparent materials, polymer-titania composites [8,9] have been developed, but their transmittances were lower than or identical to Bix(OSA-IA:7-3)-*co*-DMAA. Their radiopacity was not quantitatively evaluated but plausibly lower than bismuth-based materials due to the inherently lower radiopacity of Ti than that of Bi and the lower inorganic contents. A combination of high radiopacity with excellent transparency is a distinctive feature of Bix(OSA-IA:7-3)-*co*-DMAA. The radiopacity of bismuth carboxylate polymers examined under identical conditions almost correlates with the bismuth content (Figure S8).

The radiopacity of 0.80 mm-Al/mm-polymer for 8 keV X-ray corresponds to 0.033 mm-Pb/mm-polymer. Typical lightweight X-ray shielding goggles require 0.07 mmPb, and the thickness of the Bi27(OSA-IA:7-3)-*co*-DMAA film necessary to achieve the equivalent X-ray shielding properties is 2.1 mm, comparable to the thickness of typical protective lab goggles, which are approximately 2 mm thick. The combination of high transparency and X-ray shielding ability suggests the potential for Bix(OSA-IA:y-z)-*co*-DMAA to serve as an alternative to lead-based plastic protective goggles. However, Bix(OSA-IA:y-z)-*co*-DMAA swelled in water and therefore could not meet the standards of physical properties of lens materials. For practical applications, hydrophobic coatings are necessary for the prevention of swelling and hydrolytic degradation.

## 4. Conclusions

Lead-free polymers, Bix(OSA-IA:y-z)-*co*-DMAA, which possess both transparency and X-ray shielding abilities, were synthesized by the in situ polycondensation of a bismuth carboxylate prepolymer with a polymerizable group in DMAA, followed by photocuring. The key to obtaining the prepolymers was the intramolecular cyclization of succinate structures, which suppressed the cross-linkage in polycondensation of A_3_ and B_2_ monomers. The properties of the polymers were found to be dependent on the bismuth/organic ratios and the compositions of the two dicarboxylic acid monomers. Excellent transparency and X-ray shielding properties comparable to commercial anti-X-ray goggles indicate the potential of this material for the design of alternatives to lead-containing lenses. The next challenge is to overcome the low water resistance due to the hydrophilic components, and material designs to increase hydrophobicity are ongoing.

## Figures and Tables

**Table 1:** Synthesis of bismuth-containing polymers by polycondensation of BiPh_3_ and dicarboxylic acids.

Run	Solvent	Dicarboxylic Acid	X	Yield (%)	Unit Ratio ^a^	*D*_h_ (nm) ^b^	PDI ^b^	Solubility (THF)
1	MEK	OSA	1.0	83 ^c^	NA	440	0.31	Sol
2	THF	OSA	1.0	84 ^c^	NA	27	0.09	Sol
3	THF	OSA	1.2	73 ^c^	NA	277	0.27	Sol
4	THF	OSA	1.5	68 ^d^	NA	NA	NA	Insol
5	THF	OSA:IA	0.7/0.3	32 ^d^	NA	NA	NA	Insol
6	THF	OSA:IA	0.8/0.2	66 ^c^	NA	NA	NA	Sol to Insol
7	THF	OSA:IA	0.9/0.1	90 ^c^	92/8	152	0.25	Sol
8	THF	OSA:SA	0.5/0.5	49 ^d^	NA	NA	NA	Insol
9	THF	OSA:SA	0.7/0.3	72 ^c^	72/28	NA	NA	Sol to Insol

^a^ Determined by ^1^H NMR spectroscopy. ^b^ Measured by DLS (THF, 25 °C). ^c^ Isolated yield after precipitation into hexane. ^d^ Isolated yield after filtration of precipitate. NA: not available.

**Table 2:** Bi content, *T*_g_, and gel fraction of Bix(OSA-IA:y-z)-*co*-DMAA) with Bi content of approximately 20%.

Polymer	Bi Content (wt%) ^a^	*T*_d5_ (°C) ^b^	*T*_g_ (°C) ^c^	Gel Fraction (%) ^d^
Bi23(OSA-IA:9-1)-*co*-DMAA	23	188	89	17
Bi20(OSA-IA:7-3)-*co*-DMAA	20	190	95	59
Bi19(OSA-IA:5-5)-*co*-DMAA	19	190	100	77
Bi21(OSA-IA:3-7)-*co*-DMAA	21	196	113	78
cf. polyDMAA	0	285	89	0

^a^ Weight content of Bi determined from fed BiPh_3_ and residual DMAA. ^b^ Determined by thermogravimetric analysis (10 °C/min, N_2_). ^c^ Determined by DSC (10 °C/min, N_2_, second heating scan). ^d^ Gel fraction after Soxhlet extraction with THF for 10 h.

**Table 3:** Thermal and optical properties of Bix(OSA-IA:7-3)-*co*-DMAA) with different Bi contents.

Polymer	Bi/DMAA (mol/mol) ^a^	Bi Content (wt%) ^b^	*T*_g_ (°C) ^c^	Transmittance (% @ 589 nm) ^d^
Bi12(OSA-IA:7-3)-*co*-DMAA	7/93	12	86	91
Bi15(OSA-IA:7-3)-*co*-DMAA	10/90	15	96	90
Bi20(OSA-IA:7-3)-*co*-DMAA	15/85	20	95	90
Bi23(OSA-IA:7-3)-*co*-DMAA	18/82	23	97	90
Bi27(OSA-IA:7-3)-*co*-DMAA	25/75	27	95	90
cf. polyDMAA	0/100	0	89	92

^a^ Molar composition of Bi determined ^1^H NMR spectroscopy. ^b^ Weight content of Bi determined from fed BiPh_3_ and residual DMAA. ^c^ Determined by DSC (10 °C/min, N_2_, second heating scan). ^d^ Measured by UV-vis spectroscopy.

**Table 4:** Radiopacity and optical properties of Bix(OSA-IA:7-3)-*co*-DMAA) with different Bi contents.

Polymer	Content of Inorganic Element (wt%)	Radiopacity (mm-Al/mm-polymer)	Transmittance (% @ 420 nm) ^a^
polyDMAA	0	0.05 ^c^	90
Bi12(OSA-IA:7-3)-*co*-DMAA	12 ^b^	0.30 ^c^	90
Bi15(OSA-IA:7-3)-*co*-DMAA	15 ^b^	0.41 ^c^	89
Bi20(OSA-IA:7-3)-*co*-DMAA	20 ^b^	0.61 ^c^	84
Bi23(OSA-IA:7-3)-*co*-DMAA	23 ^b^	0.70 ^c^	85
Bi27(OSA-IA:7-3)-*co*-DMAA	27 ^b^	0.80 ^c^	83
Poly(BiMA-*co*-DMAA) [27]	18	0.60 ^c^	83
poly(BiCEA-*co*-2CEA) [28]	31	1.31 ^c^	74
Poly(MSt-*co*-TSBi) [24]	41.6–43.4	1.40–1.60 ^c^	59–68
Poly(*p*-styryldi(*p*-tolyl)bismuth-*co*-MMA) [22]	11.3	1.36 ^d^	n.d. ^e^
nano-Bi_2_O_3_-poly(vinyl chloride) [17]	36	2.29 ^f^	n.d. ^e^
Bismuth titanate-epoxy composite [18]	Bi: 46 Ti: 8	4.0 ^g^	Not transparent
Polyester-titania composite [8]	13.5	No quantitative analysis	85 ^h^
Polyurethane-titania composite [9]	ca. 3–15	No quantitative analysis	67–84

^a^ Measured by UV-vis spectroscopy. ^b^ Weight content of Bi determined from fed BiPh_3_ and residual DMAA ^c^ Cu *K*α (8.0 keV, acceleration voltage = 40 kV). ^d^ W *K*α (60.0 keV, acceleration voltage = 65 kV). ^e^ Not determined. ^f^ Calculated from mm-Pb thickness for W (40 kVp). ^g^ Calculated from mm-Pb thickness (80 kVp X-ray from a Gulmy D3150 superfical X-ray unit). ^h^ 6 μm coating on a 31 μm polypropylene sheet.

## Data Availability

All the data substantiating the conclusion of this study are included. Other primary data are available upon reasonable request from the corresponding author.

## Supplementary Materials

The following supporting information can be downloaded at https://www.mdpi.com/article/10.3390/polym17020134/s1. Figure S1: ^1^H-NMR spectrum of OSA; Figure S2: ^13^C-NMR spectrum of OSA; Figure S3: FT-IR spectra of Bix(OSA-IA:y-z)-*co*-DMAA prepared using different feed ratios of IA; Figure S4: ^1^H-NMR spectra of OSA, PolyDMAA and soluble part after Soxhlet extraction (CDCl_3_, 400 MHz); Figure S5: FT-IR spectra of Bix(OSA-IA:7-3)-*co*-DMAA prepared using different bismuth content; Figure S6: DSC profiles of Bix(OSA-IA:y-z)-*co*-DMAA) (10 °C/min, N_2_, second heating scan); Figure S7: DSC profiles of Bix(OSA-IA:7-3)-*co*-DMAA) (10 °C/min, N_2_, second heating scan); Figure S8: Relationship between bismuth composition and radiopacity of bismuth carboxylate polymers; Table S1: X-ray transmittances of Bix(OSA-IA:7-3)-*co*-DMAA films with different thickness.
