# Supplementary material for: X-Ray Shielding Polymer Based on Sequential Polycondensation of BiPh3 and Carboxylic Acids and Radical Polymerization
Source: Polymers (Basel). 2025 Jan 8;17(2):134. doi: 10.3390/polym17020134 (PMC11769120; doi:10.3390/polym17020134)
Supplement: Supplementary file 1 [file polymers-17-00134-s001.zip › polymers-3356427-supplementary.pdf]

Supporting Information for

X-ray shielding polymer based on sequential polycondensation of $BiPh_3$ and carboxylic acids and radical polymerization

Bungo Ochiai, Ryo Kamiya, Yoshimasa Matsumura, Hiroyasu Tanaka, Hideki Ueda, Kazuyoshi Uera, Kikuo Furukawa, and Yoshio Nishimura

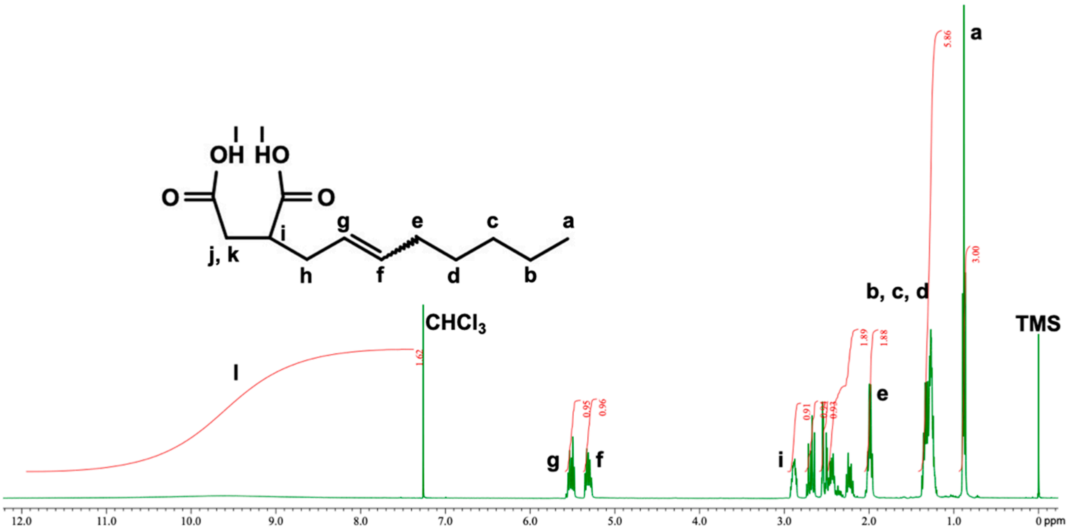

Figure S1. ^1H-NMR spectrum of OSA ($CDCl_3$, 400 MHz).

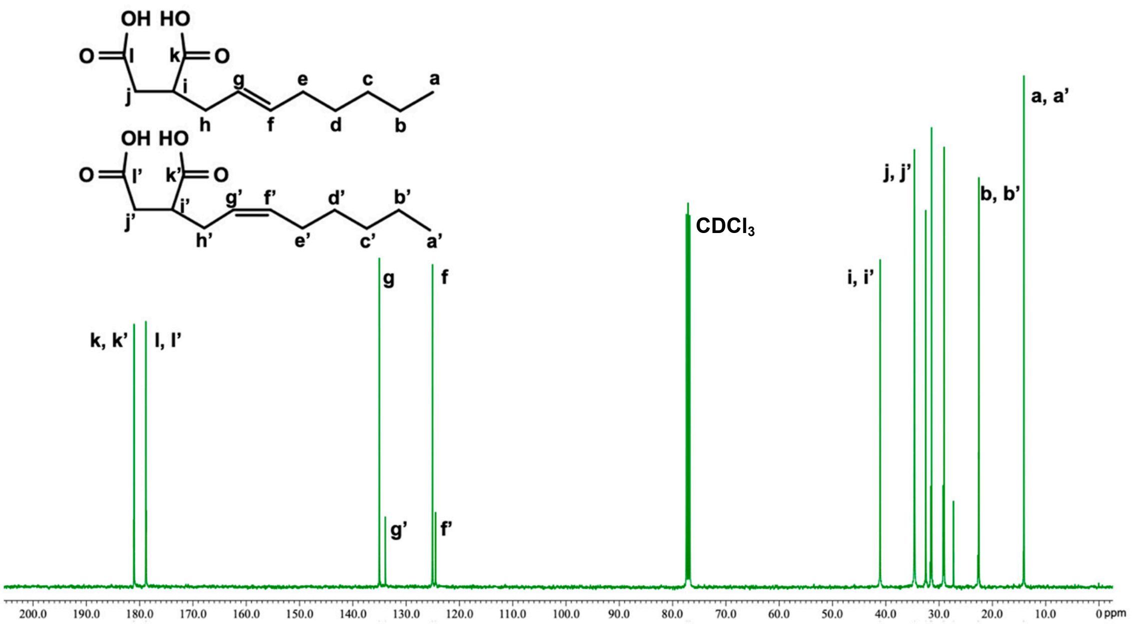

Figure S2. ^{13}C-NMR spectrum of OSA ($CDCl_3$, 100 MHz).

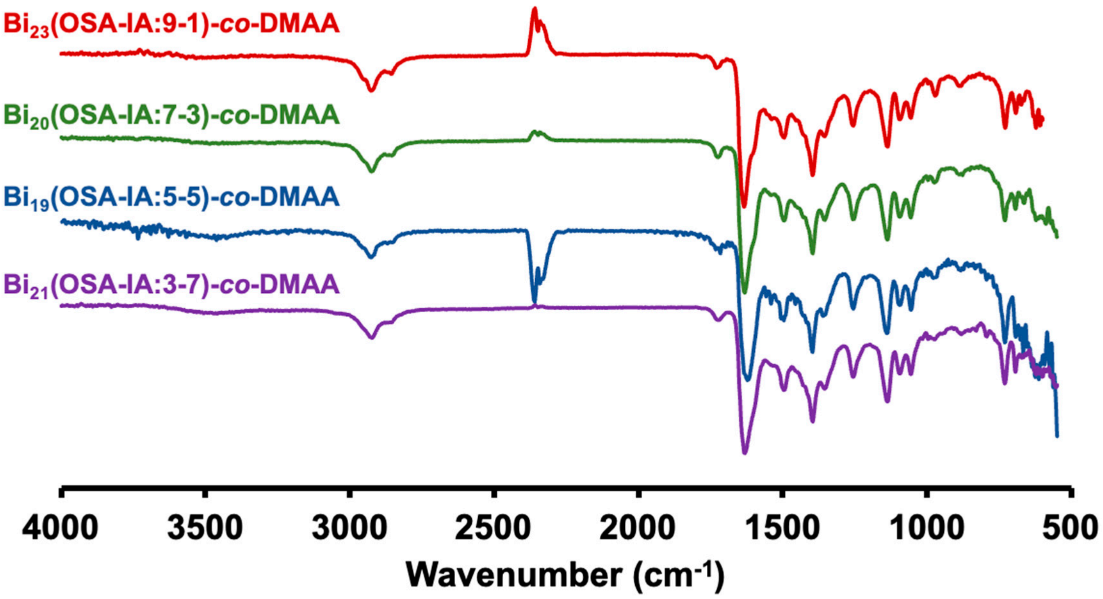

Figure S3. FT-IR spectra of Bix(OSA-IA:y-z)-*co*-DMAA prepared using different feed ratios of IA.

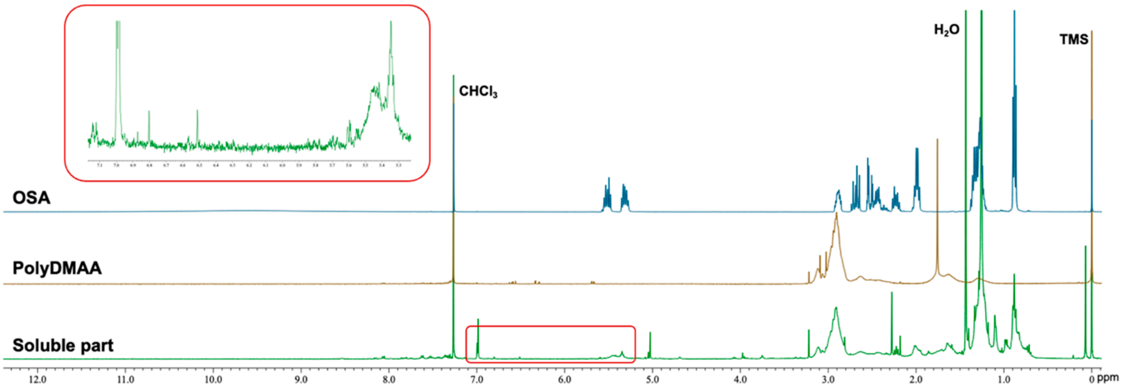

Figure S4. ^{1}H-NMR spectra of OSA, polyDMAA, and soluble parts after Soxhlet extraction ($CDCl_3$, 400 MHz).

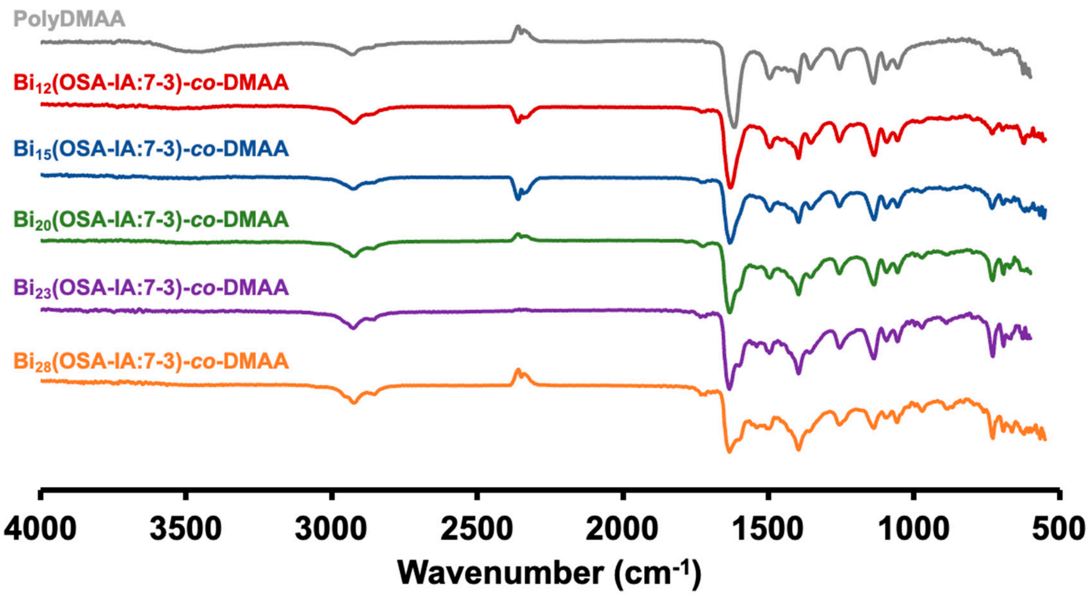

Figure S5. FT-IR spectra of Bix(OSA-IA:7-3)-*co*-DMAA prepared using different bismuth contents.

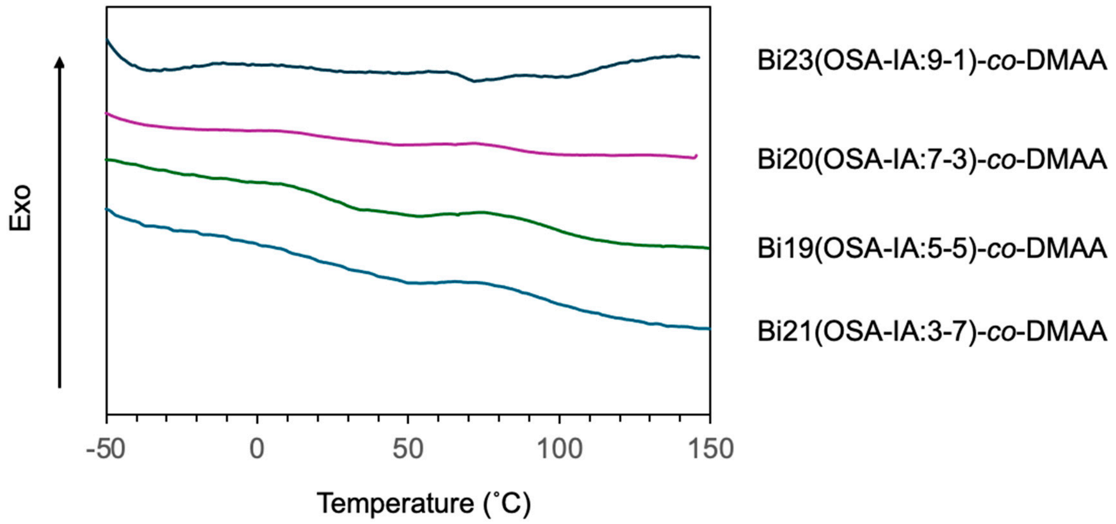

Figure S6. DSC profiles of Bix(OSA-IA:y-z)-*co*-DMAA) with Bi content of approximately 20% (10 ˚C/min, second heating scan, N_2).

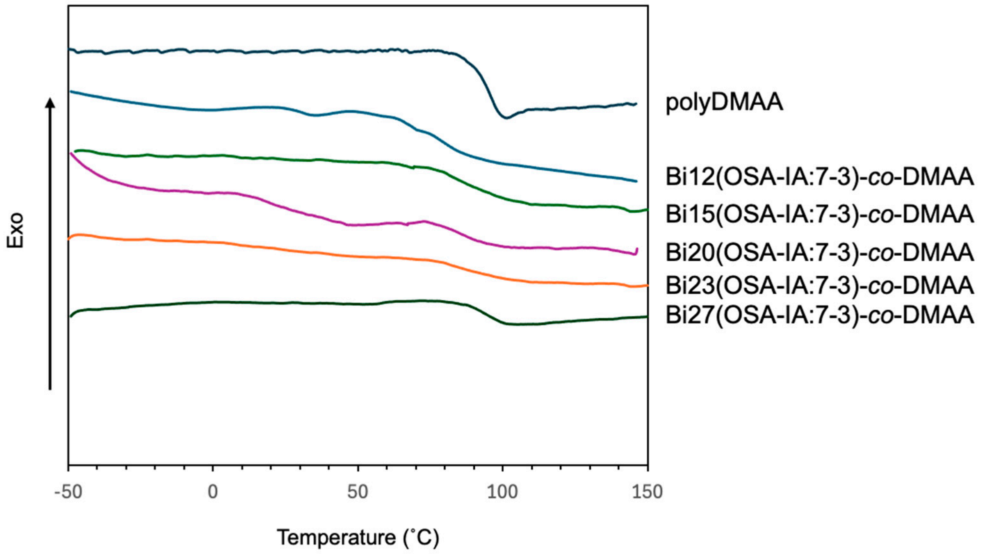

Figure S7. DSC profiles of Bix(OSA-IA:7-3)-*co*-DMAA) with different Bi contents (10 ˚C/min, second heating scan, N_2).

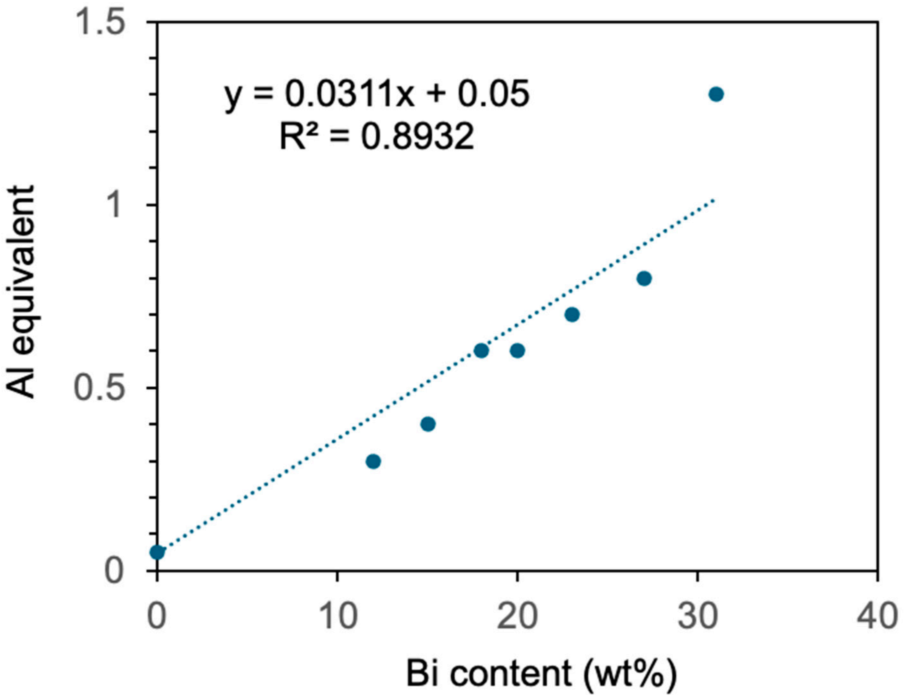

Figure S8. Radiopacity of bismuth carboxylate polymers in aluminum equivalents.

Table S1. X-ray transmittances of Bix(OSA-IA:7-3)-*co*-DMAA films with different thicknesses.

Polymer	Bi content (wt%)[a]	Thickness (μm)	X-ray transmittance (%)[b]
PolyDMAA	0	233	87.4
		507	74.6
		797	63.1
		1062	53.5
Bi12(OSA-IA:7-3)-*co*-DMAA	12	215	48.6
		401	25.8
		653	11.1
		846	5.7_8
Bi15(OSA-IA:7-3)-*co*-DMAA	15	258	30.8
		509	10.1
		711	3.9_2
		963	1.3_0
Bi20(OSA-IA:7-3)-*co*-DMAA	20	235	20.4
		504	3.3_5
		756	0.6_7
		1005	0.1_4
Bi23(OSA-IA:7-3)-*co*-DMAA	23	240	15.9
		523	2.1_3
		714	0.3_9
		1013	<0.1
Bi27(OSA-IA:7-3)-*co*-DMAA	27	246	11.3
		547	0.8_5
		723	0.2_0
		906	<0.1

[a] Weight content of Bi determined from fed $BiPh_3$ and residual DMAA [b] Cu Kα (8.0 keV), acceleration voltage = 40 kV, irradiation time = 10 min).
